# Escalating Concerns in Paediatric Hospital Care: A Qualitative Study of Involvement, Support and Empowerment of Families From Culturally and Linguistically Diverse Backgrounds

**DOI:** 10.1111/hex.70900

**Published:** 2026-10-07

**Authors:** Isis Grigoletto, Eileen Boyle, Alfizu Alhassan, Huaqiong Zhou, Sarah Cherian, Katie Browning, Joseph C. Manning, Gavin D Leslie, Fenella J Gill

**Affiliations:** ^1^ Curtin School of Nursing, Faculty of Health Sciences Curtin University Perth Western Australia Australia; ^2^ Perth Children's Hospital Child & Adolescent Health Services Nedlands Western Australia Australia; ^3^ Division of Paediatrics University of Western Australia Nedlands Western Australia Australia; ^4^ The Kids Research Institute Nedlands Western Australia Australia; ^5^ Division of Nursing Science, Pregnancy and Child Health, School of Healthcare, College of Life Sciences University of Leicester Leicester Leicestershire UK; ^6^ Nottingham Children's Hospital, Family Health Clinical Care Group Nottingham University Hospitals NHS Trust Nottingham Nottinghamshire UK; ^7^ Nursing and Midwifery Research Unit South Metropolitan Health Service, Murdoch Western Australia Australia

**Keywords:** clinical deterioration, communication, cultural diversity, family, language, limited English proficiency, migrant, paediatrics, patient safety, refugee

## Abstract

**Background:**

Families frequently recognise early signs of deterioration in their children. In English‐centric environments, families from culturally and linguistically diverse (CALD) backgrounds face challenges in escalating concerns in hospital because of language barriers, limited health literacy, uncertainty about escalation pathways, and variable staff responsiveness. Although digital approaches may support communication, it remains unclear whether they address the wider organisational conditions required for equitable escalation of care.

**Objective:**

This study explored hospital experiences of CALD families and investigated their views on a potential digital tool to escalate acute clinical concerns during their child's hospitalisation.

**Material and Methods:**

This qualitative study was guided by a multicultural consumer advisory group. Families from CALD backgrounds participated in semi‐structured focus groups and one‐to‐one interviews, conducted in English or with interpreters. Audio‐recordings were transcribed, de‐identified and analysed using reflexive thematic analysis.

**Results:**

Thirty‐four parents/carers representing 34 distinct families and 15 CALD backgrounds participated in 11 focus groups and two individual interviews. Three themes were identified: (i) communicating is not easy; (ii) shaping health system satisfaction; (iii) communicating using digital tools. Families described both supportive and challenging interactions with health staff and the system. Positive experiences were associated with plain language, clear explanations, and inclusive communication. Negative experiences included not feeling heard when raising concerns, inconsistent access to interpreters, unclear information about how to escalate concerns, and distress associated with waiting and uncertainty in emergency care. Although some families saw the value in digital tools, particularly for translation, rapid communication, and access to information, they consistently prioritised broader service changes. These included routine offers of interpreter support, clearer explanations of hospital processes, more detailed information about their child's condition and treatment, and a more welcoming environment for asking questions and raising concerns.

**Conclusions:**

Families viewed digital tools as potentially useful, but not sufficient on their own. Inequities in escalation of care were shaped not only by language discordance, but also by wider organisational conditions, including inconsistent interpreter access, unclear escalation pathways, variable staff responsiveness, and limited opportunities for families to feel heard. System change is therefore essential if digital approaches are to support equitable escalation of care in hospital settings for these families.

**Lived Experience or Public Contribution:**

A consumer and community involvement was integral to the study. A Multi‐Cultural Consumer Advisory Group (MCAG), comprising six members from CALD backgrounds (China, Egypt, India, Russia, Sierra Leone, and Vietnam), contributed to study design, recruitment planning, interpretation of findings, and refinement of the developing intervention. MCAG members also supported recruitment by sharing study information with their communities.

## 1 Introduction

Families frequently recognise early signs of deterioration in their children during hospitalisation and play a key role in communicating these concerns to health staff. In the paediatric healthcare setting, family‐centred care is a concept used to describe families being involved in their child's care and essential partners in healthcare decision‐making [1, 2]. Further, family involvement is increasingly recognised as important to timely escalation of care and patient safety. Family‐initiated escalation of care refers to processes that enable families to raise concerns about changes in their child's condition and seek further clinical review when needed. Previous research has shown that such involvement can support earlier recognition and response to clinical deterioration, although escalation is not always straightforward in practice [3, 4, 5]. Numerous approaches have been developed to incorporate families into processes for communicating their concerns about their child's condition with variable success. Communication failures, hierarchical relationships, organisational rigidity and unclear escalation processes which have capacity for cross‐cultural nuance can all limit families' ability to raise concerns and be heard [5, 6, 7].

Communication in English‐centric hospitals may be more difficult for families from Culturally and Linguistically Diverse (CALD) backgrounds. In Australia, this term generally refers to people born in countries where English is not the main language, and/or people who speak a language other than English at home [8, 9]. In hospital settings, CALD families may face additional barriers to escalating concerns, including limited English proficiency, lower health literacy, uncertainty about hospital processes, and reduced confidence in questioning staff [10, 11, 12, 13, 14]. These barriers are not only interpersonal, but they are also shaped by organisational conditions, such as whether interpreters are routinely offered, whether staff use plain language, whether escalation pathways are clearly explained, and whether families feel that their concerns will be taken seriously [15]. Delays in recognition of clinical deterioration may contribute to poorer health outcomes and reduced patient safety. In contrast, trust in staff and feeling “heard” are the key elements for success [6].

Interpreter services are an important strategy to support safe and equitable communication during hospitalisation [13, 16, 17, 18]. However, their use during urgent and emergent care can be inconsistent, delayed, or impractical in time‐critical situations [10, 19, 20]. Families from CALD backgrounds may therefore rely on ad hoc alternatives that do not fully support safe, clear and equitable communication [20, 21, 22]. This creates an important problem for escalation of care, because families need more than a way to transmit concern. They also need a service environment that is prepared to receive, understand, and respond to that concern [5, 7], to prevent and/or minimise clinical deterioration. A recent Western Australian (WA) study involving First Nations families similarly highlighted the cultural and organisational factors that can influence family escalation of care, providing complementary evidence for the importance of culturally responsive and supportive escalation systems [23].

Digital approaches, including multilingual mobile applications, have been proposed as one way to support communication between families and health staff [24, 25, 26, 27]. Their accessibility and speed may make them useful in acute settings [28]. However, it remains unclear whether digital tools can address the wider organisational conditions that shape equitable escalation of care, or whether they risk functioning as a partial response to a broader structural problem. Evidence is also limited regarding how families from CALD backgrounds experience raising concerns during their child's hospitalisation and how they perceive digital tools as a potential support. Therefore, this study explored the hospital experiences of families from CALD backgrounds and examined their views on a potential digital tool to escalate acute clinical concerns during their child's hospitalisation.

## 2 Methods

### 2.1 Study Design

This exploratory qualitative study used focus groups and one‐to‐one interviews to explore CALD families' experiences of escalating care for children in acute hospital settings. It also explored whether a digital solution might support communication and escalation of concerns, and if so, what such a solution should include. The study formed part of the broader “Find Your Voice Project,” a programme of research designed to inform development of family‐facing interventions to support escalation of care. The programme was guided by the Medical Research Council framework for developing and evaluating complex interventions [29] and drew on principles of the Experience Based Co‐Design [30].

In Australia, consumer and community involvement in health research refers to individuals as research partners who contribute lived experience and perspectives to research development, based on six key principles as defined by the National Health and Medical Research Council [31], resulting in greater translation into practice. Consumer and community involvement was integral to the study. A Multi‐Cultural Consumer Advisory Group (MCAG), comprising six members from CALD backgrounds (China, Egypt, India, Russia, Sierra Leone, and Vietnam), contributed to study design, recruitment planning, interpretation of findings, and refinement of the developing intervention. MCAG members also supported recruitment by sharing study information with their communities.

### 2.2 Setting

The state of WA has the largest geographical area served by a single health authority in the world [32]. For infants, children and young people, there is a dedicated health service. Between 2024 and 2025, the service recorded more than 1.2 million interactions with children and young people, including health assessments, outpatient appointments, and Emergency Department (ED) attendances. Families from CALD backgrounds accounted for 13.6% of these interactions. The WA Child and Adolescent Health Service (CAHS) has a Community Ambassador Programme partnering with trusted community leaders to support two‐way communication between CAHS and culturally diverse communities, improving understanding of social factors, reducing barriers, and enhancing access to services through culturally informed advice, engagement, and co‐design [33].

The ESCALATION system is an evidence‐based paediatric early warning system designed to support early recognition and response to clinical deterioration in children [34, 35]. The system was implemented across WA paediatric healthcare settings and incorporates family concern as a component. A separate family‐activated escalation of care process, called Aishwarya's CARE Call further supports families and carers when they are concerned about their child's deteriorating health condition [36].

### 2.3 Participants

Parents and carers were recruited using purposive sampling between January and September 2025 through Perth community organisations with established links to CALD communities. Eligible participants were aged 18 years or older, identified as being from a CALD background, and had experience of a child being admitted to a hospital in WA within the previous 5 years. Exclusion criteria included parents/carers of infants admitted to neonatal services and parents/carers identifying as Aboriginal and/or Torres Strait Islander descent, who represent the First Nations people of Australia [37].

### 2.4 Recruitment

Community organisations were contacted and asked to distribute study flyers inviting eligible individuals to participate in focus groups or one‐to‐one interviews. The flyers provided information about the study purpose and aims, as well as the research team's contact details for individuals interested in participating. Participants either contacted the research team directly via email or were referred by the MCAG. A researcher then assessed their eligibility, provided the participant information sheet, and explained the study aims and procedures. Following this process, focus groups or one‐to‐one interviews were arranged according to participants' preferences for time, mode of participation, and interpreter use.

### 2.5 Data Collection

#### 2.5.1 Families' Characteristics and Experience Using Digital Technology Questionnaire

Families completed a project‐specific questionnaire developed by the research team to capture families and child's characteristics and their experience using digital technology (Supporting material 1).

#### 2.5.2 Focus Groups and One‐to‐One Interviews

Data were collected through semi‐structured focus groups and one‐to‐one interviews using a study‐specific topic guide (Supporting material 2). Discussions explored families' experiences of communicating with health staff, understanding information about their child's care, raising concerns about clinical deterioration, cultural and language‐related barriers, and the use of interpreter services. Participants were also asked about their views on a potential multilingual digital approach to support escalation of concerns, including its acceptability, accessibility, preferred mode of delivery, and potential barriers to use. These discussions were moderated by an experienced female PhD‐prepared qualitative researcher from a CALD background, who had no prior relationship with the families. Accredited interpreters were used where required (interpreters certified by the National Accreditation Authority for Translators and Interpreters ‐ NAATI ‐ in Australia). Focus groups were conducted online via Microsoft Teams and audio‐recorded separately to support data quality and participants' privacy. Data were collected until no new relevant information, themes or insights were obtained and data saturation was reached [38]. Field notes were collected during focus groups and interviews.

Data were collected until no new relevant information or insights were identified and data saturation was considered to have been reached.

### 2.6 Data Analyses

Families' characteristics were summarised descriptively using IBM SPSS Statistics, version 29.0. Audio‐recordings were transcribed verbatim by a qualified transcriber and de‐identified. NVivo 15 (Lumivero, 2023) was used to support data management and organisation. Field notes were reviewed after each discussion and analysed iteratively, so that ideas identified in earlier discussions could be explored in later discussions.

Qualitative data were analysed using Braun and Clarke's six‐phase reflexive thematic analysis [39]. Analysis was iterative and interpretive rather than consensus‐driven. Two researchers (IG, AA) read and re‐read transcripts, generated initial codes, and developed candidate patterns across the dataset. Field notes were used to support interpretation and to inform subsequent data collection. A third researcher (EB) contributed to reflexive discussions that helped refine subthemes and themes. Preliminary findings were summarised in an infographic and shared with the MCAG to support discussion and interpretation of the findings. The infographic included a study overview and key preliminary findings relating to barriers to raising concerns and the use of interpreter services from both family and health staff perspectives (Supporting material 3).

### 2.7 Ethics

The study was approved by the Child and Adolescent Health Service (RGS6020) and the University's (HRE2023‐0412) Human Research Ethics Committees. Written informed consent was obtained from all participants, using interpreters as required. The study is reported in line with the Consolidated Criteria for Reporting Qualitative Research checklist [40].

## 3 Results

A total of 11 community sites were contacted for participant recruitment (Figure 1). Ninety‐one individuals expressed interest in participating. Eight were subsequently found to be ineligible because they resided outside WA. Of the remaining individuals, 49 declined to proceed with participation following subsequent contact by email or telephone. Specific reasons for non‐participation were not collected. The final sample of 34 participants comprised 31 parents and 3 carers, each representing a distinct family, who collectively reported experiences relating to 49 children.

**Figure 1:**
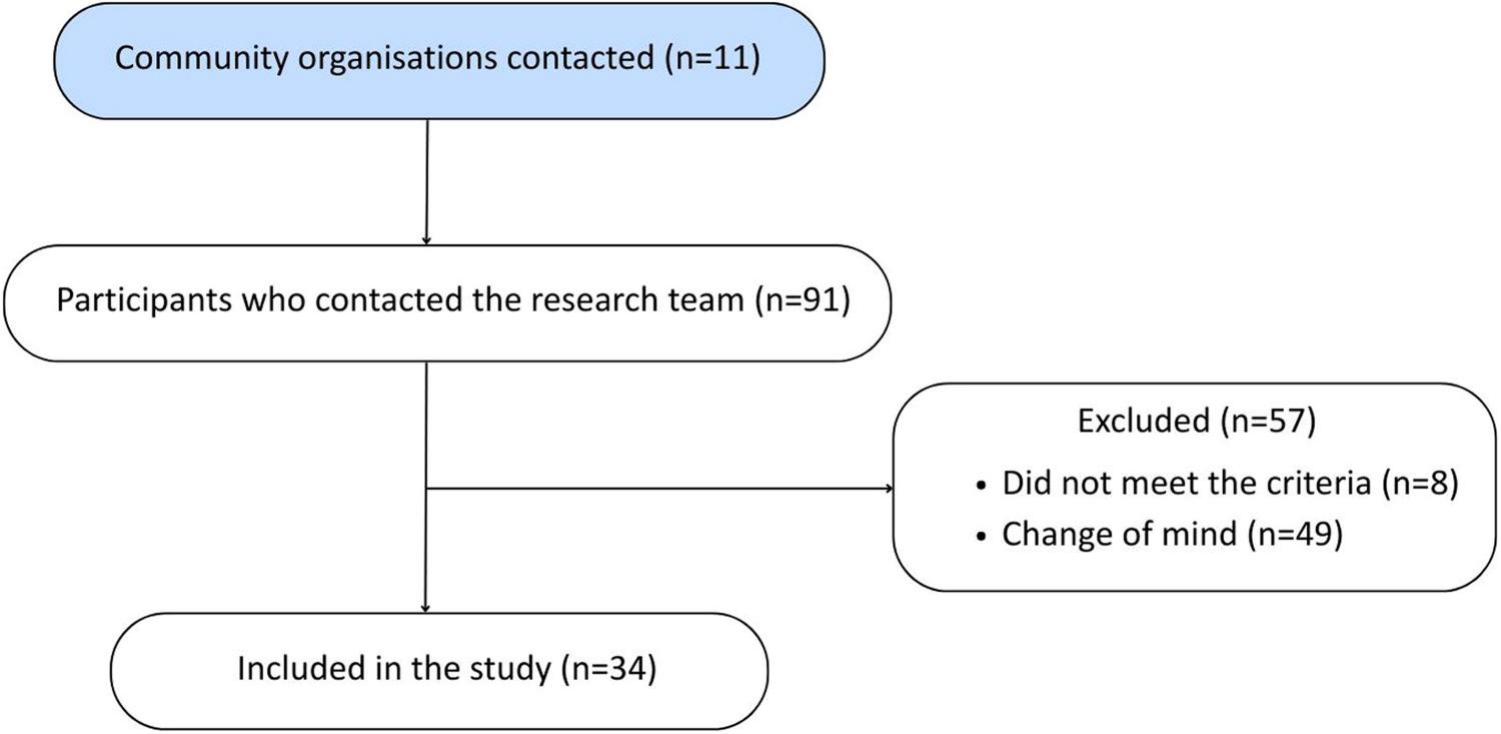
Recruitment flow chart of the study.

Thirty‐four CALD parents and carers participated in 11 singles focus groups (number of participants ranged from three to seven) and two participated in single one‐to‐one interviews. The focus groups lasted between 46 and 60 min, and the one‐to‐one interviews lasted 19 and 23 min. Two focus groups (nine participants, 27%) of Afghani and Iranian backgrounds were facilitated with certified NAATI interpreters. Participants were parents/careers of 49 children and represented 15 cultural and linguistic groups. Most were female (82%), aged 30–49 years (91%) and had attended university (52%). They described experiences across seven WA hospitals. Participant characteristics are shown in Table 1.

**Table 1:** Characteristics of participants and children.

Variable			
Relationship with child (*n* = 34)			
Parent			31 (91%)
Friend			3 (9%)
Age (yr), *n* (%) (*n* = 33^a^)			
18–29			3 (9%)
30–49			30 (91%)
Gender, *n* (%) (*n* = 33^a^)			
Male			6 (18%)
Female			27 (82%)
Highest level of schooling, *n* (%) (*n* = 33^a^)			
Did not attend school			3 (9%)
Primary school			5 (15%)
Secondary school			4 (12%)
Diploma			4 (12%)
University			17 (52%)
Country of birth, *n* (%) (*n* = 33^a^)			
Afghanistan			9 (27%)
Vietnam			6 (18%)
India			3 (9%)
Others*			15 (45%)
Language spoken at home, *n* (%) (*n* = 33^a^)			
Pashto			6 (18%)
English			5 (15%)
Vietnamese			6 (18%)
Others^#^			16 (48%)
Time living in Australia, (yr) (*n* = 33^a^)			
> 10			20 (61%)
5–10			9 (27%)
1–4			4 (12%)
Time speaking English, (yr), *n* (%) (*n* = 33^a^)			
From birth			1 (3%)
> 10			18 (55%)
5–10			5 (15%)
1–4			5 (15%)
< 1			2 (6%)
Fluency, *n* (%) (*n* = 33^a^)			
Excellent/like native speaker			6 (18%)
Very well			12 (36%)
Quite well			8 (24%)
Not very well			6 (18%)
Not well at all			1 (3%)
How well understand doctor^c^, *n* (%) (*n* = 33^a^)			
Understand everything without an interpreter			14 (42%)
Understand most aspects without an interpreter			11 (33%)
Understand only limited aspects and needs an interpreter			3 (9%)
Need an interpreter for all conversations			5 (15%)
Use of internet, *n* (%) (*n* = 33^a^)			
Yes			29 (88%)
No			4 (12%)
Use of internet difficulty, *n* (%) (*n* = 29)			
Very easy			17 (59%)
Easy			10 (35%)
Neither easy nor difficult			2 (7%)
Mobile phone access internet, *n* (%) (*n* = 33)			33 (100%)
Children (*n* = 49)			
Times child hospitalised, *n* (%) (*n* = 47^b^)			
Once			13 (28%)
Twice			8 (17%)
Three times			8 (17%)
≥ Four times			4 (9)
ED visit only			14 (30%)
Child's age, *n* (%) (*n* = 47^b^)			
< 3 months			1 (2%)
3 months – 1 year			4 (9%)
1–4 years			14 (30%)
5–11 years			20 (43%)
> 12 years			8 (17%)
Child with current medical condition, *n* (%) (*n* = 47^b^)			16 (34%)
Child born overseas, *n* (%) (*n* = 49)			34 (69%)

*Note:* Yr: years, m: male, n: sample, %: percentage, ED: emergency department. ^a^: 33 participants completed the questionnaire, ^b^: data were available for 47 children, ^c^: full description in Supporting material 1 – question 16, *: Brazil, Cameroon,z China, Eritrea, Ethiopia, Iran, Mauritius, Papua New Guinea, Russia, Sierra Leone, Taiwan, Tanzania, #: Arabic, Brazilian Portuguese, Creole, French, Fulah, Gujarati, Russian, Swahili, Telugu.

### 3.1 Focus Groups and Interviews Findings

Analysis generated 38 codes, 11 subthemes and three overarching themes: (i) communicating is not easy; (ii) shaping health system satisfaction; and (iii) communicating using digital tools (see Figure 2). While many reported positive interactions with health staff, their accounts consistently showed that equitable escalation of care depended not only on language support, but also on whether families were informed, listened to, and able to navigate hospital systems with confidence. Across all themes, participants described escalation of care as shaped by wider organisational conditions, including communication practices, interpreter access, responsiveness from staff, and clarity of hospital processes. Although digital tools were seen as potentially helpful, they were rarely viewed as sufficient on their own.

**Figure 2:**
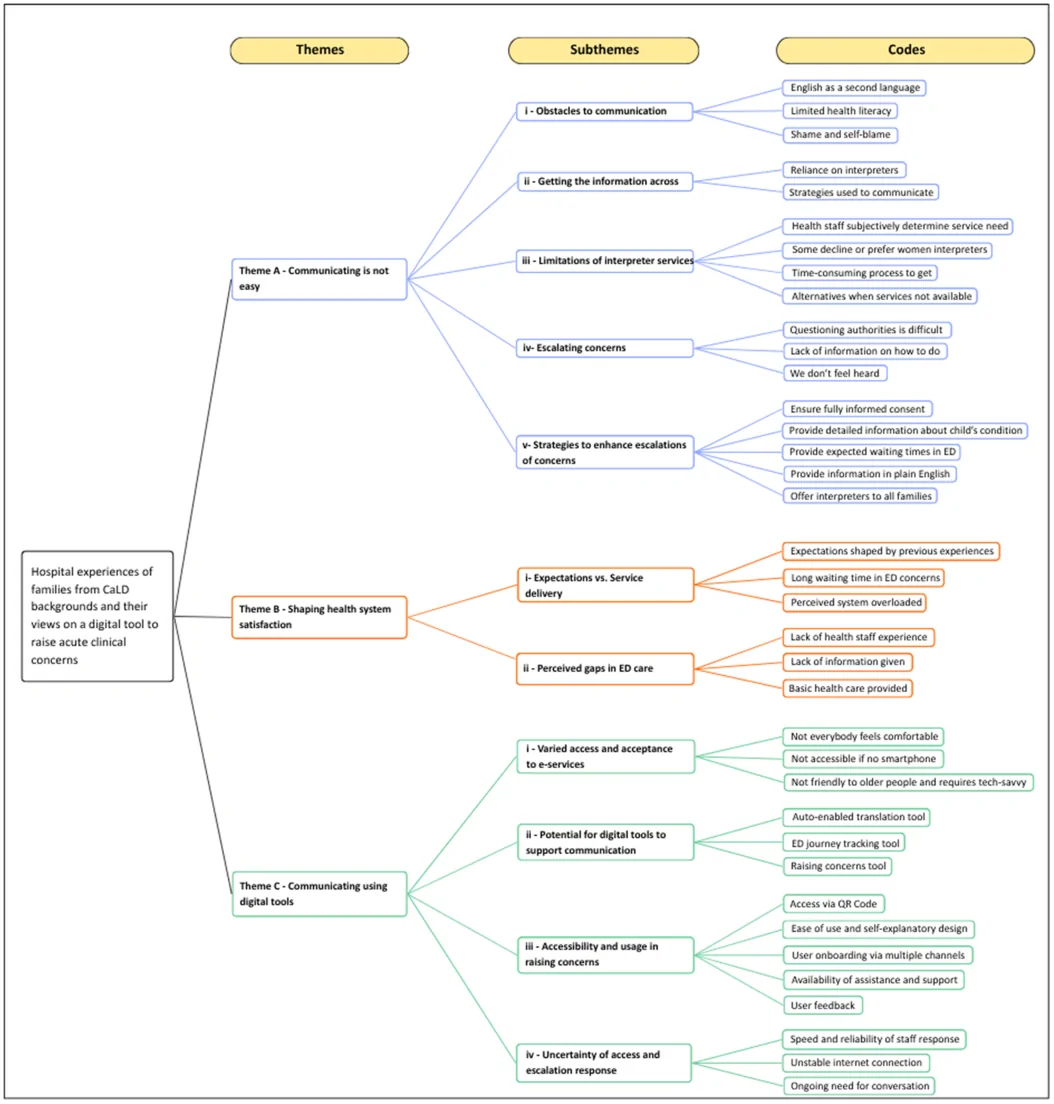
Themes, subthemes and codes. CALD, culturally and linguistically diverse; ED, emergency department.

### 3.2 Theme A—Communicating Is Not Easy

This theme captures the barriers families from CALD backgrounds faced when trying to communicate, raise concerns, and navigate hospital care. Participants described escalation as difficult not only because of language barriers, but also because they were not always told how to raise concerns, did not always feel heard when they did so, and experienced inconsistent access to interpreters. Families also identified practical changes that would make escalation easier and safer.

### 3.3 Subtheme i—Obstacles to Communication

Many families reported positive and pleasant experiences with health staff. Communication with staff was influenced by families' level of English fluency with many experiencing difficulties due to their self‐reported limited English and/or limited health literacy, particularly related to understanding medical terminology. Conversely, the inability to communicate proficiently often led families to report feeling ashamed and to perceive themselves as being responsible for communication difficulties.

So, um, sometimes, um, probably it's hard for me because of them, um, specific terms and, um, diagnosis, um, specific terms and, um, diagnosis. But in, in general I was feeling quite, quite confident and staff were supporting.

(FG 11)

The doctor was like—he doesn't understand me. Uh, if he can't understand me, then he won't help me …was like, just do something. Do something to help my child…so, I, I, I tried…, to help, and I didn't get the help I deserve. I don't know if I should blame the doctor or if I should blame myself.

(FG 4)

### 3.4 Subtheme ii—Getting the Information Across

Families reported how interactions and conversations related to health procedures frequently relied on interpreters. Families also described strategies they and health staff used to facilitate communication when interpreters were not available. These included use of body gestures and non‐verbal communication and/or health staff providing information in plain (basic) English, limiting engagement and safety.

I needed an interpreter, you know, to, um, uh, make things easier for me.

(FG 1)

Even if my children or my husband would be there, we would still need the interpreter. We would ask for interpreter.

(FG 13 – facilitated by an interpreter)

I had to find all possible ways to make them understand me. That was why I writing everything on paper, to get them to read it through, to understand better, to be able to help my son life.

(FG 1)

They're very nice. If, if we wouldn't understand so they will, um, make an understand in the simpler way, more easier.

(FG 12 – facilitated by an interpreter)

### 3.5 Subtheme iii—Limitations of Interpreter Services

Families with limited English proficiency reported they relied on interpreters for their interactions and conversations with health staff. Some families reported interpreter services were offered when staff subjectively identified limited English or cultural markers (e.g., traditional clothing). In contrast, families who were perceived to speak good English described not being offered interpreter services.

But there's no asking us whether we need to have interpreter.” I don't think I got any questions escalated, uh regarding to that…Yeah, maybe they see me speaking English.

(FG 7)

I think most of them ask in receptions [if we want an interpreter].

(FG 13 – facilitated by an interpreter)

The services were sometimes declined when families felt confident in their own level of English fluency. Some families also considered that using a translation application on their phone was sufficient or preferred to rely on a family member to interpret, or refused the service due to concerns about their privacy, particularly when the interpreter was from the same cultural community.

Because my husband was with me, and he speaks English, so it was easy for me. If my husband wasn't present, so it was going to be very difficult for me.

(FG 13 – facilitated by an interpreter)

Um, I never, I never considered to have one…Mmm. Cause I have my phone.

(FG 8)

Yeah, for me like it's invading my privacy because if I don't ask for an interpreter and you come and sit there and they do not explain to me what's happening, if I don't need one, if I needed one I'll ask, but if I don't need one, I don't want someone else to come and come and listening to what's happening to my child. If I, I don't, I don't want it, I'm not comfortable with that.

(FG 10)

Women from some cultures were not comfortable to have a male interpreter when discussing specific health conditions or situations, such as strategies to improve breastfeeding. In these cases, women stated that the only option was to have a female interpreter to support these conversations or, if this was not possible, to rely on their husbands to interpret and explain the information to them.

That would be good to have female interpreter…No other choice [if no women interpreter available to discuss specific health conditions].

(FG 13 ‐ facilitated by an interpreter)

A commonly reported challenge was the time‐consuming process involved in accessing an interpreter in emergencies, when communication support was needed urgently. For some rare dialects, families noted it could take several days to get an interpreter.

It was up to, uh, two weeks for me to, you know, get the interpreter…

(FG 1)

The problem is because there are not many [language name] interpreters. When I go to the appointment, I have an appointment at 2:00, and then by the time we arrive 2:00, and then the interpreter leaves. They say there is no interpreter. Another 30, 40 min, it takes time that they will get another [language name] interpreter.

(FG 13 ‐ facilitated by an interpreter)

Families also described using alternative communication strategies when interpreters were not available. These included communicating through written notes, seeking health staff who spoke the same language, using online translation tools and artificial intelligence platforms to support communication (e.g., Google Translate and ChatGPT), and asking siblings or other family members to interpret.

…there is ChatGPT that it, it's very helpful…In emergency time.

(FG 12 — facilitated by an interpreter)

She's saying she's happy that her son would do interpreting for her.

(FG 13 — facilitated by an interpreter)

### 3.6 Subtheme iv—Escalating Concerns

Escalating concerns about their child's health was described as challenging. Several factors influenced whether and how families escalated concerns about their child's condition. These included limited English literacy, as previously presented in subtheme (i), cultural norms, perceived lack of information about how to escalate concerns, and not feeling heard when they attempted escalation.

…I tried to explain to him that my son has been here for a week now but there is no improvement. I haven't seen any changes. I kinda just tried to do some signs, you know.

(FG 4)

So I don't know if it was the miscommunication or what was the story. But the cultural, uh, I think it's one of the, um, yeah, facets of this story. It's a cultural thing (not questioning).

(FG 11)

But at no point, when I went, did someone tell us, even when they didn't know I was a doctor.” “Oh, you know, if you're not happy, this is who you can contact… (to raise concerns about your child's condition).

(FG 7)

### 3.7 Subtheme v—Strategies to Enhance Escalations of Concerns

Families identified strategies that could help them navigate the hospital and its procedures, and consequently, feel more able and comfortable to escalate concerns. The need for a welcoming hospital environment, which involves supporting and empowering families to escalate concerns, underpinned perceived success. Suggested strategies included: i) ensuring fully informed consent around treatment and procedures; ii) providing more detailed and descriptive information about their children's conditions and treatment offered, reflecting the practice of “inappropriate simplification” of messaging where interpreters were not provided and/or communication was rushed; iii) providing clear information in the ed about expected waiting times; iv) providing information in plain English, so that families feel included in the system and better able to understand how to navigate hospital facilities and procedures; v) offer interpreter services to all families, to avoid assumptions about their level of English fluency and to ensure that all families are aware of and able to access interpreter support.

And those basic principles should be giving time to actually understand the diagnosis, reinforcing and asking, Do you have any more questions for me? Is there anything else? Would you like me to go through with this?

(FG 7)

Waiting is fine. I understand like they have more patience, but at least if you answer our question and at least it's little bit ease for us to grasp and sit patiently.

(FG 6)

This is what you need to look for if you go here and there. I need help, press interpreter. Press or I don't know, something else. Just a couple of those major, uh, things that they need help with…

(FG 11)

### 3.8 Theme B—Shaping Health System Satisfaction

This theme shows how families' satisfaction with care was shaped by more than clinical treatment alone. Experiences were strongly influenced by waiting, uncertainty, communication quality, and the extent to which staff explained what was happening. For many participants, dissatisfaction reflected not only interpersonal problems, but also pressures within the service environment, particularly in emergency care.

### 3.9 Subtheme i—Expectations Versus Service Delivery

Families' previous experiences in their home countries shape their expectations of the health system. Those from countries they perceived as having a functional health system reported higher expectations of the Australian health system.

…It's just, uh, the lifestyle here is just slow pace…in [home country], everything's so efficient…For me, it's not about language. It's about how, how the system goes.

(FG 8)

Some migrant background participants appeared more critical than families from countries with less structured systems or from regions affected by resource scarcity or war, who conversely appeared more positive and satisfied with the care they had received.

Yes, especially with the number of hospitals around my area in that country, and here, it's, like, different. We won't find, like, the many hospitals here, like when it's a [sic] emergency.

(FG 6)

Experiences in ED were the main source of dissatisfaction and were often interpreted as experiences that negatively influenced families' views of the health system. Long waiting times were widely reported as a key difficulty. From the families' perspective, wait times caused delays they attributed to an overloaded system with negative consequences for their mental health.

Like, they treat me and everything is good, but the only problem is here, that, the waiting time in the hospital emergency department is very long time…

(FG 6)

I think that waiting period can actually cause a lot of mental distress.

(FG 5)

As this mother explained her experience waiting in the ED for 7 h for her child to be seen by a doctor:

Can you please explain me what, what are the steps or what's happening or when the doctor is coming at least? Can you tell me? And they say, like, we can't tell you anything at the moment. You have to wait. You have to wait.

(FG 6)

### 3.10 Subtheme ii—Perceived Gaps in ED Care

Some families reported what they perceived to be lack of experience by ED staff, described in terms of receiving insufficient information about treatment processes, and their views of inadequate basic health care provided.

The other thing I see, there's a lotta junior staff that, like, 90 percent of the people you see, they're all junior. And, like, it, sometimes, takes a long time to get a, like, a‐a s‐a senior doctor, someone that actually know, better, what's goin’ on.

(FG 8)

Um, so that's, that's a vacuum of information which causes a slight annoyance because we are in an emergency to get treated and to know what's the issue, and we are in the time of not really knowing, uh, what just happened and how do we go about it and such.

(FG 5)

I can't question it [treatment procedures]. It's their decision, though, but… I found that really interesting, like…basic, uh, investigations…that took us [a] really long [time] to get it out.

(FG 5)

### 3.11 Theme C—Communicating Using Digital Tools

This theme captures participants' views on the possible role of a digital tool and comprises four subthemes related to technology use in health services, and the potential digital tools in supporting that could be implemented in hospitals to support communication and involvement in the escalation of concerns. Although some participants saw value in digital tools, the requirements for digital approaches, especially for translation and escalating concerns, would only be useful if embedded within a wider system that offered clear information, ease of access and timely staff response.

### 3.12 Subtheme i—Varied Access and Acceptance to e‐Services

Importantly, not all families felt comfortable or eager to use technology in their daily lives with some participants explaining how they avoided using additional smartphone applications whenever possible. Smartphone access was not universal, plus there was the identified need for a level of digital literacy to use e‐services in healthcare. Certain cohorts (e.g. older parents/carers, especially those with visual impairments) were also identified as having additional barriers to using communication e‐solutions.

…at the moment I start hating apps. I start stopping.'Cause everywhere…It's something like if there is a way not to use an app…

(FG 11)

For me, I would like to say not, not every parent maybe have access to technology, especially in certain situations or demographic, or demographic or some potential barrier, including the digital device or the, let's say the age or ability.

(FG 2)

### 3.13 Subtheme ii—Potential for Digital Tools to Support Communication

Some families that were comfortable using healthcare technology suggested application‐based tools could be used to improve communication between families and health staff. An auto‐enabled digital translation tool, which could provide a rapid translation in urgent situations, was viewed as a helpful and less time‐consuming tool that could help staff explain hospital and treatment procedures and may mitigate waiting for long periods to access interpreters. An ED journey tracking tool, to provide information on expected waiting times, and their child's position in the queue to see a doctor was suggested to reduce the stress associated with waiting.

It's [auto‐enabled translation tool] actually helpful'cause it could be different languages. Like me, a translator will be helpful.

(FG 4)

[ED journey tracking tool] … it will keep the patient informed in terms of how long it'll be taking for them to be treated. I think, I feel that could be a very engaging, assuring way, if you're looking the digital way that's something you should get.

(FG 5)

Views were divided about the value of a digital tool to support communication escalation of concerns. Some considered it may be helpful in stressful situations where their second language [i.e., English] may “disappear” or be less proficient under pressure.

…it's good to be good because you know, even, even if you speak English, but when you have problem sometime even the language disappear, so it be, it'll be very good for, to help the parents, while in there.

(FG 9)

Families explained how a digital tool may help staff more easily identify when non‐English proficient families were concerned about their child's health. On the other hand, some families did not consider this type of technology‐based solution necessary, believing they it should not be required to have a specific tool, but the health service be skilled enough to meet the needs of all families.

That's it. You don't need, even need an app. So if you want just to speak to that person and they understand you. So I wouldn't say that an extra app is needed for that purpose only.

(FG 11)

### 3.14 Subtheme iii—Accessibility and Usage in Raising Concerns

Families emphasised what was important for a digital tool was that it should be easy to access (using a QR Code rather than an app) and to navigate.

Yeah, QR code, everyone knows about QR codes.

(FG 5)

Pictorial representations, animations and information both written and audio were viewed as key elements to improve engagement and support understanding for those families with limited education, literacy and/or health literacy.

Those with a picture or infographic should help press the right button. Um, a solution, what I always say is pictures, I mean, all languages I'm not really sure if you would be able to get, but pictures, videos, which are more explanatory in terms of its actions, um, any of these things would be a universal approach…

(FG 5)

Because some um, people, so they don't even have the education of reading and writing in their own language. And also having a visual and audio, that's great as well.

(FG 12 — facilitated by an interpreter)

Families also highlighted the importance of being informed about the opportunity to use a digital tool before being at the hospital, a time when families are often under considerable stress.

…so it's good to get it before because while you, while you are like, you're not stressed, it's easy to, to take the information.

(FG 9)

Suggested strategies to be informed included advertising availability of the digital tool on television, introducing it during general practitioner (GP) appointments, and promoting it within communities.

So you can advertise in school, it's a part of government area. Or like libraries, community centre, where all the other different community are meeting. So that's the best place for display.

(FG 6)

To ensure a positive user experience, families recommended including a guide on how to use the digital tool and making assistance available for those with limited digital literacy.

…and what you can have is you can at least have some sort of summary as to when that button [referring to the digital tool to escalate concerns] should be used. So when people get there, at least it's there for them to see and read. You know, maybe two, three major points that if you see this, this or this, press this button, you know?

(FG 5)

Families reported that provision of feedback on experiences and usability of a digital tool should be requested when families were no longer under stress and worried about their child's health.

… (ask feedback on the tool) maybe one or 2 days later, then you can just check with them if you have their numbers. Email address or something.

(FG 6)

### 3.15 Subtheme iv—Uncertainty of Access and Escalation Response

Families expressed concerns about potential issues or barriers to using a digital tool such as limited internet connection may interfere with its usability and uncertainty about timely response by staff.

I'll be concerned, yeah, if…if it's working with network, and, you know, there is bad network, yeah, definitely, um, it will be, uh, one of the barriers.

(FG 4)

Importantly the key concern identified was that direct English communication with health staff (following escalation) was still required for parents to be able to explain their concerns, underpinning the critical need for safe communication at all times.

…You're activating a response to a sick kid. But then, when you activate a response, we have to be able to communicate the concerns. So, automatically, I think, if you're activating the response and the nurse comes and looks at the child, but ask us, “Why are you worried?” they need, we need to be able to have that translation capability right there and now.

(FG 7)

Collectively, these accounts suggest that participants did not view digital innovation as a substitute for equitable service design, but as a possible adjunct within a broader package of system‐level supports.

## 4 Discussion

In this study, families from CALD background described escalation of care as shaped not only by language barriers, but also by wider organisational conditions, including inconsistent interpreter access, unclear escalation pathways, variable staff responsiveness, and limited opportunities to feel heard. Although digital tools were viewed as potentially helpful, particularly for translation and access to information, participants did not see them as sufficient on their own. Instead, families consistently prioritised broader service changes that would make communication, navigation, and escalation of concerns more equitable in practice [10, 11, 12, 13, 14]. Our findings extend this literature by showing that, for CALD families, the problem is not only one of language discordance. It is also a question of whether the service is organised in ways that enable concerns to be expressed, recognised, and acted on. In this sense, digital tools may support communication at a single point in the escalation pathway, but they cannot substitute for equitable service design.

Engaging with the health system was often difficult for CALD families, even when individual interactions with staff were positive. Families described barriers linked to limited English proficiency, health literacy, uncertainty about hospital processes, and discomfort in questioning staff. These findings are consistent with previous research showing that families using languages other than English may be less able to participate fully in care, ask questions, and raise concerns [14, 15, 41]. For better engagement in the system and their child's care, families often relied on interpreter services, to ensure clear and accurate communication with health staff [10]. Previous studies and national safety standards acknowledge that interpreter services remain an important strategy for reducing these barriers and improving communication [10, 13, 15, 16, 17, 18, 42, 43]. However, our findings also show that interpreter access was inconsistent and sometimes dependent on staff assumptions about language need. This is important because equitable escalation of care depends not only on the availability of interpreters, but on whether communication support is offered routinely, promptly, and in ways that families find acceptable.

Family escalation of care is a relational process rather than a purely technical one [6, 7, 14]. Families are more likely to raise concerns when they feel informed, respected, and taken seriously [7, 14]. When acute clinical concerns are promptly escalated, deterioration can be recognised earlier and potentially prevent further decline, resulting in better outcomes [4]. In our study, saliently participants reported that they were not always told how to escalate concerns and did not always feel heard when they did so. These findings suggest that inequities in escalation are produced not simply by communication difficulty, but by whether the service actively legitimise family voices, including those from CALD and/or non‐English proficient backgrounds.

Families' satisfaction with the health system and care received, is shaped by their involvement in their child's care [2], communication with health staff [10, 16], ability to navigate hospital procedures and amenities [10], understand of child's health condition and proposed treatment [10]. Adding to our understanding of family centred care, we found families' previous experiences in their home countries appeared to influence their satisfaction with the system. People from resource‐scarce settings and/or trauma backgrounds (e.g. refugee‐like experiences) tended to express greater satisfaction than those from countries they perceive as having a high‐functioning health system.

Participants identified several ways in which digital tools might support communication, including rapid translation, easier access to information, and reduced uncertainty while waiting in emergency care. However, they also made clear that digital support would have limited value unless embedded within broader system change [44]. A digital tool may help a family signal concern, but it cannot by itself ensure that the concern is understood, taken seriously, or acted on.

Participants identified several ways in which digital tools might support communication, including rapid translation, easier access to information, and reduced uncertainty while waiting in emergency care. However, they also made clear that digital support would have limited value unless embedded within broader system change. A digital tool may help a family signal concern, but it cannot by itself ensure that the concern is understood, taken seriously, or acted upon. Digital innovation should therefore be understood as one component of a broader approach to equitable escalation of care rather than as a stand‐alone solution.

### 4.1 Strengths

This study focused on the voices of CALD families, including those with limited English language proficiency who are typically excluded from qualitative research processes, yet are a substantial proportion of public hospital users in WA. This enabled perspectives that are often under‐represented in research on hospital communication and escalation of care to be included. The sample also included families from 15 cultural and linguistic backgrounds, which strengthened the breadth of experiences captured. A further strength was the integration of consumer and community involvement throughout the study, which supported the relevance and acceptability of the research design and interpretation. The range of opinions and solutions voiced challenged thinking that digital solutions are a panacea for clinical deterioration and family engagement. Rather required organisations to consider basic approaches to families feeling welcomed and safe in hospital environments, which builds the foundations of trust with health professionals.

### 4.2 Implications for Clinical Practice and Policy

These findings have several implications for clinical practice and policy. Health staff should consider routinely providing clearer explanation of escalation pathways, routine encouragement to ask questions, and more responsive communication from staff, offering professional interpreter support rather than relying on assumptions about families' English proficiency, clearer information about hospital processes, and a more welcoming environment for asking questions and raising concerns. Digital tools may support communication and raise concerns but should be implemented as an adjunct, rather than a substitute to approaches already in place.

### 4.3 Limitations

The study focused on the voices and perceptions of CALD participants within a state that is very multicultural and is a key strength. As such, all translating findings to other marginalised population groups may not be directly possible which is a limitation. Whilst provision of interpreters was made, social disability bias is a further limitation, noting that those participants with greater social connectivity and higher English proficiency were more likely to participate in the study than those who required interpreters and/or were recent arrivals. It is possible that the experiences of more socially isolated families, or those with the greatest communication barriers, are under‐represented. Third, because participants could report experiences of care across different hospitals from up to 5 years previously, recall bias is possible, and differences in timing and setting may have influenced how experiences were recalled and interpreted. Fourth, although online participation reflected families' preferences, families with limited digital access, connectivity, or confidence may still have been under‐represented. Finally, recruitment through community networks and voluntary self‐selection may have favoured families with greater social connectivity, English proficiency or capacity to participate. Consequently, families experiencing the greatest communication, social or access barriers may be under‐represented, and the findings may understate some of the barriers experienced by these families.

## 5 Conclusion

Families from CALD backgrounds described escalation of care as shaped not only by language barriers, but also by wider organisational conditions, including inconsistent interpreter access, unclear escalation pathways, variable staff responsiveness, and limited opportunities to feel heard. Although digital tools were seen as potentially helpful for translation, information access, and raising concerns, they were not viewed as sufficient on their own. Our findings suggest that digital approaches should not be implemented in isolation. System change is essential to ensure that CALD families are routinely informed, supported, and able to escalate concerns within a service that is prepared to receive and respond to those concerns equitably across all encounters.

## Author Contributions

**Isis Grigoletto:** investigation, writing – original draft, methodology, visualisation, writing – review and editing, formal analysis, project administration, data curation. **Eileen Boyle:** conceptualisation, methodology, visualisation, writing – review and editing, formal analysis. **Alfizu Alhassan:** formal analysis, writing – review and editing. **Huaqiong Zhou:** writing – review and editing, visualisation. **Sarah Cherian:** writing – review and editing, conceptualisation, investigation, funding acquisition, visualisation. **Katie Browning:** writing – review and editing, investigation, conceptualisation, visualisation. **Joseph C Manning:** writing – review and editing, conceptualisation, investigation, visualisation. **Gavin D Leslie:** writing – review and editing, conceptualisation, funding acquisition, visualisation, supervision. **Fenella J Gill:** writing – review and editing, resources, supervision, conceptualisation, investigation, funding acquisition, visualisation, project administration.

## Ethics Statement

The study was approved by the Child and Adolescent Health Service (RGS6020) and the University's (HRE2023‐0412) Human Research Ethics Committee. Written informed consent was obtained from all participants, using interpreters as required.

## Consent

The authors have nothing to report.

## Conflicts of Interest

The authors declare no conflicts of interest.

## Supporting information


Supporting File 1

Supporting File 2

Supporting File 3

## Data Availability

The data that support the findings of this study are available from the corresponding author upon reasonable request. The data presented in this study are available upon reasonable request. All data relevant to the study are included in the article or uploaded as Supporting Information.
