# Supplementary material for: Escalating Concerns in Paediatric Hospital Care: A Qualitative Study of Involvement, Support and Empowerment of Families From Culturally and Linguistically Diverse Backgrounds
Source: Health Expect. 2026 Oct 7;29(5):e70900. doi: 10.1111/hex.70900 (PMC13643952; doi:10.1111/hex.70900)
Supplement: Supplementary file 1 [file HEX-29-e70900-s001.pdf]

Government of Western Australia
Child and Adolescent Health Service

Find Your Voice Study

Families with linguistically and diverse characteristics raising concerns about their child: involve, support and empower

Curtin University

PROUDLY SUPPORTED BY telethon 7

Study overview

89 health staff and 34 families from Culturally and Linguistically Diverse backgrounds (CaLD) interviewed

5 different health staff roles

15 different CaLD backgrounds

FIND YOUR VOICE

Preliminary key findings

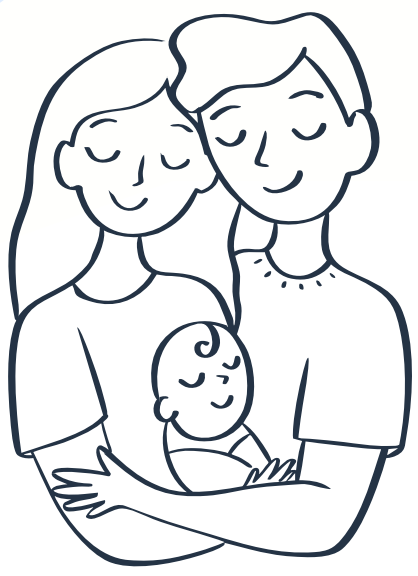

According to CALD families, why don't they raise concerns?

- Difficulty understanding medical terms.
- Limited information on how to raise concerns.
- Shame of communicating due to english level
- Some don`t feel heard.

What do CaLD families say about using interpreter services?

- Some aren't confident in their English level.
- Sometimes service is not offered.
- Arranging the service takes time.
- Some cultures prefer female interpreters.

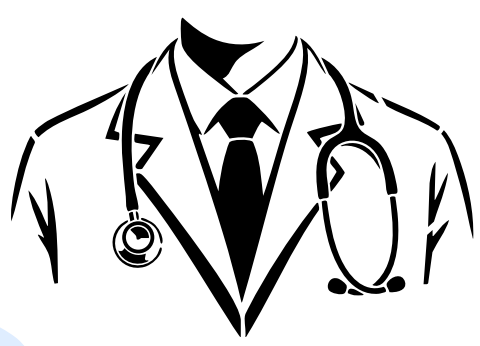

To what do health staff attribute CaLD families' difficulty in raising concerns ?

- Difficulty understanding medical terms.
- Limited English and health literacy levels.
- Some cultures do not share health concerns openly.

According to health staff, what are challenges using interpreter services?

- There appear to be not enough interpreters available .
- Staff are sometimes unsure about when to call an interpreter
- There is limited time to talk with interpreters.
- Some staff feel unsure about how accurate the interpretation is.
- Arranging an interpreter can take a lot of time.

Thank you to everyone who has contributed to the 'Find Your Voice" study.

www.escalation.com.au
