# Supplementary material for: Escalating Concerns in Paediatric Hospital Care: A Qualitative Study of Involvement, Support and Empowerment of Families From Culturally and Linguistically Diverse Backgrounds
Source: Health Expect. 2026 Oct 7;29(5):e70900. doi: 10.1111/hex.70900 (PMC13643952; doi:10.1111/hex.70900)
Supplement: Supplementary file 2 [file HEX-29-e70900-s002.pdf]

Supplementary material 2

Parent/Carer (Interview/Focus Group Guide)

PART 1:

Q1. How did you find talking with staff when your child was at hospital?
Prompts:
- Australian Hospitals
- Hospitals in your country

Q2. How well did you understand what was going on/was the plan for medical treatment?
Prompts:
- Australian Hospitals
- Hospitals in your country

Q3. How did you feel about answering questions and giving your opinion to hospital staff about your child's health?
Prompts:
- Emotional or stressful interactions (shame, confidence, offense, not feel listened, cultural norms around questioning authority)
- Cultural differences in communication
- Use of interpreters
- Strategies to ensure understanding

Q4. Were you ever worried that your child was getting sicker in hospital?

- If so, can you please tell me more about that experience?
  Prompts:
  - What did you do?
  - Did you say you were worried?
  - Did you feel comfortable speaking about that?
  - What happened?
  - Is there anything that made, or might have made saying you were worried easier or more difficult?

- If not, can you please think about what you might have done in that situation and why?

Q6. What suggestions would you give to make the hospital system more welcome to you and other parents from a CaLD background?
Prompts:
- How to improve communication?
- What strategies could calm you when in a stressful situation that you don't feel heard.

Q6. Do you have other ideas or opinions about these questions that we have not already talked about?
If interpreter needed for the focus group:

Q1. Did you use an interpreter when your child was in hospital?

Q2. How do you / would you tell staff you were worried about your child if there was no interpreter available?

Q3. Are you confident and/or have you been able to ask hospital staff for an interpreter to discuss your concerns?

PART 2:

We think it would be helpful to have an online or digital solution/way available in many languages, for families from different cultures and language background to tell hospital staff if they think their child is getting sicker.

This would **not** be meant as a replacement for interpreting services and talking with staff, but as an extra way to raise concerns, for example, if an interpreter is not immediately available or if you are still worried after talking with staff or feel unable to talk with staff.

We would really appreciate your honest opinion about this.

Q1. If it was available, would you use it - why/why not?

Q2. What would make you more likely to use this digital solution? And less likely to use it?

Q3. How do you think we could delivery this digital solution?
Prompts:
- App – need to download
- QR Code – don't need to download

Q4. What is the best way to tell you and other parents about this digital solution?
Prompts:
- Nurses
- Allied Health
- Clerk on admission
- Visual communication -> communication through patients' entertainment, posters, SMS. Or a media or campaign?
- Communication through the specialists (before they come to the hospital)? E.g. chronic conditions, etc.

Q5. Do you feel there are any barries to use digital solution in hospital? Can you please explain why?

Q6. How do you think this digital solution would influence your child hospitalization?
Prompts:
- Positive aspects: feel heard, more confident, better outcomes for your child, discharged, pre quickly,
- Negative aspects: more stressful, more confusing, technology would be a problem

Q7. Do you have other opinions or thoughts about this idea that we have not already talked about?
