# Supplementary material for: Escalating Concerns in Paediatric Hospital Care: A Qualitative Study of Involvement, Support and Empowerment of Families From Culturally and Linguistically Diverse Backgrounds
Source: Health Expect. 2026 Oct 7;29(5):e70900. doi: 10.1111/hex.70900 (PMC13643952; doi:10.1111/hex.70900)
Supplement: Supplementary file 3 [file HEX-29-e70900-s003.pdf]

Supplementary material 1

Participants' characteristics Questionnaire

Q1. Do you need an interpreter to help you fill out this form?

☐ Yes ☐ No

Q2. What is your relationship to the child?

☐ Parent
☐ Extended family (aunt/uncle/cousin)
☐ Carer
☐ Friend
☐ Other (please state): ________________

Q3. What is your gender? (Please select the option that best describes you, including your pronouns):

☐ Woman (she/her)
☐ Man (he/him)
☐ Non-binary (they/them)
☐ Prefer to self-describe (please include your pronoun): ______________
☐ Prefer not so to say

Q4. What is your age?

☐ 18 – 29 years old
☐ 30 – 49 years old
☐ 50 – 69 years old
☐ More than 69 years old

Q5. What is your highest level of schooling?

☐ I did not attend school
☐ Primary school (e.g. ages 4 - 11)
☐ Secondary school (e.g. ages 11 - 16)
☐ Senior secondary school (e.g. ages 16 - 18)
☐ Diploma (e.g. vocational or trade certificate)
☐ University

Q6. Which hospital (s) have you attended with your child in the last 5 years?

☐ Perth Children's' hospital
☐ Fiona Stanley Hospital
☐ Joondalup Hospital
☐ Private hospital in Perth
☐ West Australian Regional hospital
☐ Bunbury hospital
☐ Busselton hospital
☐ Albany hospital
☐ Kalgoorlie hospital
☐ Narrogin hospital
☐ Port Hedland hospital
☐ Broome hospital
☐ Fitzroy Crossing hospital
☐ Other: ____________________________

Q7. What is the number of times your child/children have been to a hospital (emergency department or in-patient ward) in WA in the last previous 12 months?

☐ Once
☐ Twice
☐ Three
☐ More than three

Q8. What is the current age of your child/children?

☐ Less than 3 months
☐ 3 months – 1 year old
☐ Over 1 year old – 4 years old
☐ 5 – 11 years old
☐ 12 and above years

Q9. Does your child/any of your children have a medical condition (illness, injury, disorder or health issue)?

☐ Yes ☐ No If yes, please specify which one: ________________________

Q10. In which country was your child/children born? (please state): ___________________

Q11. In which country were you born? (please state): ___________________

Q12. What language do you speak at home? (please state): ___________________

Q13. How many years have you been living in Australia?

☐ From birth
☐ More than 10 years
☐ 5 - 10 years
☐ 1 – 4 years
☐ Less than a year

Q14. How long have you been speaking English?

☐ From birth
☐ More than 10 years
☐ 5 - 10 years
☐ 1 – 4 years
☐ Less than a year

Q15. How fluent do you speak English?

☐ Excellent / Like a native speaker
☐ Very well
☐ Quite well
☐ Not very well
☐ Not well at all

Q16. Which of the following options best describes how well you understand your doctor? (Please select one option only).

☐ Option 1. I understand **everything** that is explained in a medical conversation (diagnosis, mec testing and follow up) and can answer questions and express my opinion **without needing an interpr**

☐ Option 2. I understand **most aspects** of medical conversations and can answer questions and ex opinion **without needing an interpreter.**

☐ Option 3. I understand **only limited aspects** of medical conversations and struggle to answer ques express my opinion **without an interpreter**.

☐ Option 4. **I need an interpreter for all** medical conversations.

Q17. Do you use the internet (for example, to look up information and speak with people)?

☐ Yes ☐ No

If yes, do you find this:

☐ Very easy
☐ Easy
☐ Neither easy nor difficult
☐ Difficult
☐ Very difficult

Q18. Does your mobile phone allow you to access the internet?

☐ Yes ☐ No
